# Preoperative muscle weakness as defined by handgrip strength and postoperative outcomes: a systematic review

**DOI:** 10.1186/1471-2253-12-1

**Published:** 2012-01-17

**Authors:** Pervez Sultan, Mark A Hamilton, Gareth L Ackland

**Affiliations:** 1 Department of Anesthesia, Stanford University Medical Center, Stanford, California 94305, USA; 2 Anaesthesia and Critical Care, St Georges Hospital NHS Trust, London, UK; 3 Centre for Anaesthesia, Critical Care and Pain Management, University College London, London, UK; 4 Department of Medicine, University College London, London, UK

## Abstract

**Background:**

Reduced muscle strength- commonly characterized by decreased handgrip strength compared to population norms- is associated with numerous untoward outcomes. Preoperative handgrip strength is a potentially attractive real-time, non-invasive, cheap and easy-to-perform "bedside" assessment tool. Using systematic review procedure, we investigated whether preoperative handgrip strength was associated with postoperative outcomes in adults undergoing surgery.

**Methods:**

PRISMA and MOOSE consensus guidelines for reporting systematic reviews were followed. MEDLINE, EMBASE, and the Cochrane Central Register of Controlled Clinical Trials (1980-2010) were systematically searched by two independent reviewers. The selection criteria were limited to include studies of preoperative handgrip strength in human adults undergoing non-emergency, cardiac and non-cardiac surgery. Study procedural quality was analysed using the Newcastle-Ottawa Quality Assessment score. The outcomes assessed were postoperative morbidity, mortality and hospital stay.

**Results:**

Nineteen clinical studies (17 prospective; 4 in urgent surgery) comprising 2194 patients were identified between1980-2010. Impaired handgrip strength and postoperative morbidity were defined inconsistently between studies. Only 2 studies explicitly ensured investigators collecting postoperative outcomes data were blinded to preoperative handgrip strength test results. The heterogeneity of study design used and the diversity of surgical procedures precluded formal meta-analysis. Despite the moderate quality of these observational studies, lower handgrip strength was associated with increased morbidity (n = 10 studies), mortality (n = 2/5 studies) and length of hospital stay (n = 3/7 studies).

**Conclusions:**

Impaired preoperative handgrip strength may be associated with poorer postoperative outcomes, but further work exploring its predictive power is warranted using prospectively acquired, objectively defined measures of postoperative morbidity.

## Background

A substantial minority of patients sustain an excess of postoperative complications [1] and accelerated, post-hospital discharge mortality [2]. In surgical procedures known to have a mortality of greater than 5% in the UK, elderly patients (mean age 75 years) and emergency procedures account for over 80% of deaths but less than 15% of total procedures [3]. Physician- and patient-friendly, practical and inexpensive tools are required to guide and risk-stratify perioperative management objectively for this cohort of patients. Measurements of exercise capacity and muscle strength are associated with increased all-cause and cardiovascular mortality in the general population [4-7]. However, the comprehensive assessment of cardiovascular reserve - most objectively using cardiopulmonary exercise testing [8] - is challenging for immobile patients, time-consuming, and costly to extend as a general screening tool to the wider, at-risk surgical population. By contrast handgrip strength is an inexpensive, objective bedside test which has established population norms [9-13] and has been extensively tested in a range of chronic general medical conditions [14]. It may reflect, in part, the association of impaired muscle strength with malnutrition [15] and cardiopulmonary or metabolic diseases [4-7]. Hand grip strength can be assessed by instructing the patient to keep their shoulders adducted and neutrally rotated, the arm in a vertical position, the wrist in a neutral position and to squeeze the grip with maximal strength. The highest result in a seated or semi-seated position may be used [16,17]. Whether a robust relationship between preoperative handgrip strength and postoperative outcomes exists is unclear, since variable, and frequently retrospective, definitions of postoperative morbidity have been employed as outcome measures [18]. Therefore, we performed a systematic review of the literature to ascertain if preoperative assessment of handgrip strength is associated with (i) postoperative morbidity, (ii) length of hospital stay.

## Methods

**Figure 1:**
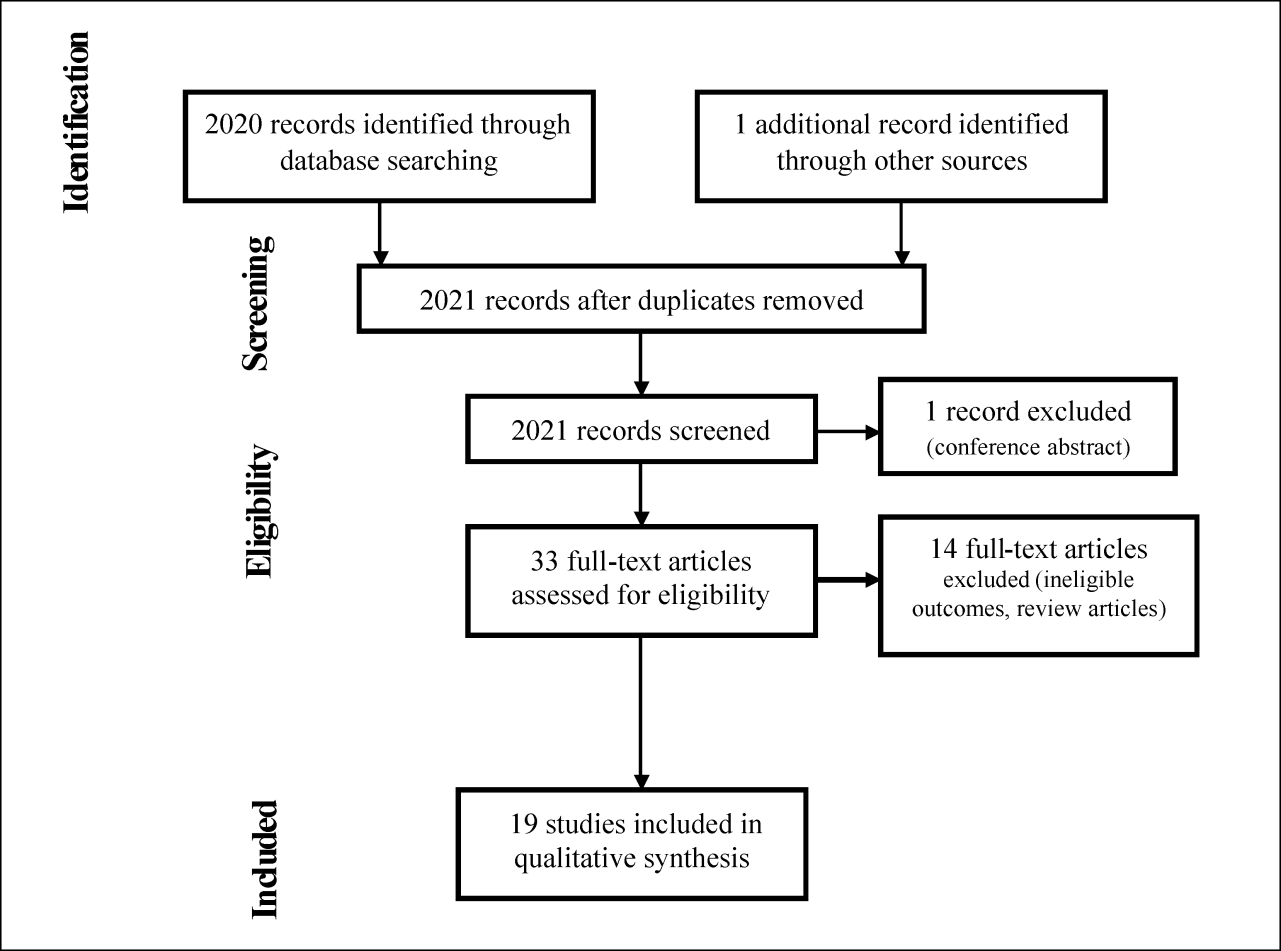
**Flow diagram showing systematic review synthesis, in accordance with PRISMA guidelines**.

The systematic review was undertaken in accordance with the PRISMA [19] (Preferred Reporting Items for Systematic reviews and Meta-Analyses) and MOOSE (Meta-analysis of Observational Studies in Epidemiology) [20] guidelines. Figure 1 summarizes the flow of information through the different phases of this systematic review. A checklist demonstrating adherence to the PRISMA guidelines is available online (Additional File 1).

Two of the authors (P.S. and M.A.H.) searched the electronic databases MEDLINE, EMBASE, and the Cochrane Central Register of Controlled Clinical Trials independently using the following population search terms: 'postoperative complications' OR 'perioperative complications' OR 'surgical complications' OR 'surgical outcome'. These search results were combined with 'handgrip dynamometry' OR 'hand grip dynamometry' OR 'hand grip strength' OR 'handgrip strength' OR 'maximal voluntary contraction' in the title or abstract text using the Boolean search operator 'AND'. (Maximal voluntary contraction is the term used most commonly in the literature to describe maximal force produced by a muscle as it contracts while contracting against the hand dynamometer). The references of retrieved articles were hand searched for any relevant articles not identified in the original search. The study selection criteria were limited to include only studies reported in the English language and those involving human adults undergoing surgery (including cardiac and transplant surgery). Each abstract was screened to identify studies that had assessed handgrip strength prior to surgery. Studies were excluded if postoperative outcomes focussed on upper limb neuromuscular functional outcomes alone.

The data were extracted on to a standardized data entry form by each reviewer. Differences between the reviewers were resolved by re-examination of the original manuscript until consensus was obtained. Data extracted for comparison included year of publication, primary author, total number of subjects, mean patient age, proportion of male subjects and co-morbidity (where reported). The method of quantifying or qualifying handgrip strength was recorded.

The specific outcomes sought in each article were: (i) mortality, (ii) postoperative morbidity, categorized according to the Post Operative Morbidity Survey, (iii) length of hospital stay [21]. Primary and/or secondary outcomes were recorded according to the a priori intention of each original article. Each outcome was evaluated qualitatively according to either qualitative and/or quantitative assessment of handgrip strength. Because there were a limited number of studies with homogenous design for each outcome, a meta-analysis could not be performed.

The procedural quality of each trial was assessed using several criteria, although no studies were excluded on the basis of these assessments. The quality of studies was scored according to the Newcastle-Ottawa Quality Assessment Scale [22] (Additional File 2), on a scale from 1 (poor) to 8 (excellent), which includes patient follow-up rates as a marker of study quality. Disagreements about the eligibility of a study or differences between the two sets of information extracted were resolved through discussion between all authors. After abstraction of information, a level of evidence was assigned to the outcomes of each study. Two authors (P.S. and M.A.H.) independently reviewed and scored each study using this method.

## Results

Nineteen studies were identified that compared postoperative outcomes in relation to handgrip strength (Table 1), comprising 2194 patients [9-13,16,17,23-34]. A wide range of surgical sub-specialties was explored. Four studies were conducted in patients undergoing urgent surgery for hip fractures. One study explored the effect of pre-operative nutritional supplementation on grip strength [25]. Although supplementation improved post-operative grip strength compared to the control group, it was not related to patient outcome. Only two studies ensured that investigators who evaluated postoperative morbidity also remained blinded to the pre-operative grip strength values [10,17]. A wide range of exclusion criteria were reported between studies. One-third of studies reported the patient drop-out rate.

**Table 1:** Basic demographics, defined primary/secondary outcomes and handgrip site used for patient studies.

Author	Year	Study type	Surgery (urgency/type)	Number of patients (n)	Age (mean ± SD or mean [range])	Gender (% male)	Primary Outcome	Secondary Outcome	Handgrip: dominant vs. non-dominant?
Beloosesky [16]	2010	Cohort*	Urgent fractured neck of femur	105	81 ± 7	31	Functional outcome	Not stated	Dominant
Wehern [23]	2005	Cohort	Urgent Hip fracture	205	81 ± 8	0	Functional outcome	Not stated	Right arm
Mahalakshmi [10]	2004	Case control	Elective general	100	42[13-70]	62	Complications	Not stated	Non dominant
Cook [17]	2001	Case control	Elective CABG	200	Not stated	73	Complications	Not stated	Both hands
Figueiredo [24]	2000	Cohort	Elective Liver transplant	53	50 ± 12	59	Complications	Not stated	Both hands
Le Cornu [25]	2000	Case control	Elective liver transplant	82	24-68	73	Complications	Not stated	Not stated
Visser [26]	2000	Cohort	Urgent Hip fracture	90	79 ± 8	0	Mobility	Not stated	Not stated
Guo [11]	1996	Case control	Elective oral and maxillofacial cancers	127	54 ± 15	69	Complications	Not stated	Non dominant
Watters [27]	1993	Cohort	Elective general	40	< 50 y group (36 ± 9) > 70 y group (77 ± 5)	65	Relate Muscle strength to body composition and nitrogen balance	Not stated	Non dominant
Schroeder [34]	1993	Cohort	Elective general	84	54 ± 18	44	Post-op fatigue	Not stated	Dominant
Griffith [28]	1989	Cohort	Elective general and vascular	61	66^#^[41-82]	75	Complications	Not stated	Dominant
Kalfarentzos [12]	1989	Case control	Elective general	95	70 [42-88]	56	Complication	Not stated	Not stated
Brenner [29]	1989	Cohort	Elective general and vascular	249	Not stated	66	Complications	Not stated	Not stated
Webb [30]	1989	Case control	Elective general	90	58 [20-88]	60	Complications	Not stated	Not stated
Shukla [31]	1987	Case control	Elective Major general	110	20-70	49	Complications	Not stated	non dominant
Hunt [13]	1985	Case control	General, Orthopedic, Urology, Gynaecology, Cardiovascular, Endocrine and Miscellaneous	205	45 ± 17	46	Complications	Not stated	Not stated
Davies [32]	1984	Cohort	Urgent Fracture neck of femur	76	Not stated	Female	Complications	Not stated	Not stated
Klidjian [33]	1982	Case control	Elective general	120	60 [24-86]	55	Complications	Not stated	Non dominant
Klidjian [9]	1980	Case control	Elective general	102	57 [16-81]	46	Complications	Factors impairing handgrip strength	Non dominant

*Retrospective study; # median value.

The majority of studies measured handgrip strength pre-operatively (Table 2). Eleven studies did not comment on how long before surgery the handgrip strength was measured [9,10,17,24,25,28,30-33]. Guo et al did not comment on whether handgrip strength was measured pre or post-surgery [11]. Very few studies achieved a quality assessment score less than 6, consistent with moderate quality (Table 3, Additional File 1).

**Table 2:** Timing of handgrip measurements in patient studies.

Author	Year	Timing of measurement
Beloosesky [16]	2010	7-10 days and 1, 3, 6 months post-op
Wehern [23]	2005	During hospitalisation and 2, 6, 12 months post-op
Mahalakshmi [10]	2004	Pre-op- timing not specified
Cook [17]	2001	Pre-op- timing not specified
Figueiredo [24]	2000	Pre-op- timing not specified
Le Cornu [25]	2000	Pre-op- timing not specified
Visser [26]	2000	2-10 days and 12 months following admission
Guo [11]	1996	Not specified whether pre or post-surgery
Watters [27]	1993	Pre-op on day of surgery and post-op days 2, 4 and 6
Schroeder [34]	1993	Pre-op on day of surgery
Griffith [28]	1989	Pre-op- timing not specified
Kalfarentzos [12]	1989	2-3 days pre-op
Brenner [29]	1989	2 days pre-op
Webb [30]	1989	Pre-op- timing not specified
Shukla [31]	1987	Pre-op- timing not specified
Hunt [13]	1985	12-72 hours pre-op
Davies [32]	1984	Pre-op- timing not specified
Klidjian [33]	1982	Pre-op- timing not specified
Klidjian [9]	1980	Pre-op- timing not specified

**Table 3:** Newcastle -Ottawa Quality Assessment Scores (NOS score).

				Selection				Comparability	Outcome		
Study	Year	Study type	NOS score	1	2	3	4	1	1	2	3
Beloosesky [16]	2010	Cohort	6	D	a*	a*	a*		b*	a*	a*
Wehren [23]	2005	Cohort	6	a*	a*	b*	a*		b*	a*	c < 60%
Figueiredo [24]	2000	Cohort	6	D	a*	a*	a*		b*	a*	a*
Visser [26]	2000	Cohort	6	D	a*	a*	a*		b*	a*	a*
Watters [27]	1993	Cohort	7	a*	a*	b*	a*		b*	a*	a*
Schroeder [34]	1993	Cohort	6	D	a*	b*	a*		b*	a*	a*
Griffith [28]	1989	Cohort	7	a*	a*	b*	a*		b*	a*	a*
Brenner [29]	1989	Cohort	6	D	a*	b*	a*		b*	a*	a*
Davies [32]	1984	Cohort	7	a*	a*	b*	a*		b*	a*	a*

Mahalakshmi [10]	2004	Case-control	7	a*	a*	b*	a*		b*	a*	a*
Cook [17]	2001	Case-control	7	a*	a*	b*	a*		b*	a*	a*
Le Cornu [25]	2000	Case-control	7	a*	a*	b*	a*	a*	c	a*	a*
Guo [11]	1996	Case-control	6	a*	a*	b*	a*		c	a*	a*
Kalfarentzos [12]	1989	Case-control	6	a*	a*	b*	a*		c	a*	a*
Webb [30]	1989	Case-control	4	C	b	b*	a*		c	a*	a*
Shukla [31]	1987	Case-control	6	a*	a*	b*	a*		c	a*	a*
Hunt [13]	1985	Case-control	4	C	b	b*	a*		c	a*	a*
Klidjian [33]	1982	Case-control	4	C	b	b*	a*		c	a*	a*
Klidjian [9]	1980	Case-control	4	C	b	b*	a*		c	a*	a*

Letters represent answer for corresponding numbered question in each section. A study can be awarded a maximum of one star for each numbered item within the Selection and Outcome categories. A maximum of two stars can be given for Comparability. See Additional File 1 for full details of assessment criteria.

### Definition of impaired handgrip strength

Variable definitions for impaired handgrip strength have been used across studies (Table 4). Studies compared values of grip strength obtained from healthy controls, reference populations or patients who did not sustain postoperative morbidity with surgical patients. For example, 9 studies defined impaired handgrip strength as < 85% of a general, age-matched population - but these reference populations were not common between studies. Table 1 demonstrates that six studies measured handgrip strength exclusively from the non-dominant hand, compared to 3 studies that measured handgrip strength in the dominant hand. Seven studies did not report which hand was tested. 11/19 studies did not report the timespan over which handgrip strength measurements preceded surgery. Variable time points were used between studies to assess postoperative handgrip strength. Detailed protocols for the performance of handgrip strength were absent in the majority of studies.

**Table 4:** Definitions used for impaired handgrip Strength.

Author	A priori definition of Impaired handgrip strength?	Definition of impaired handgrip strength	Post-hoc Definition/comparison
Beloosesky [16]	NO		Functional Independence Measure 6 months postoperatively
Wehren [23]	NO		Activities of Daily Living
Mahalakshmi [10]	YES	< 85% control values	
Cook [17]	NO		According to low or high risk status
Figueiredo [24]	NO		Critical Care length of stay
Le Cornu [25]	NO		< 85% and > 85%
Visser [26]	NO		Loss in grip strength post-operatively
Guo [11]	YES	< 85% control values	
Watters [27]	NO		Loss in grip strength post-operatively
Schroeder [34]	NO		Post-operative fatigue
Griffith [28]	NO		Loss in grip strength post-operatively
Kalfarentzos [12]	YES	< 85% control values	
Brenner [29]	NO		
Webb [30]	YES	< 85% population norm	
Shukla [31]	NO		< 85% and > 85%
Hunt [13]	YES	< 85% healthy controls	
Davies [32]	NO		< 15 kg
Klidjian [33]	YES	< 85% controls [1980 study]	
Klidjian [9]	NO		< and > 85%

### Postoperative morbidity

Table 5 summarizes the 15 studies that detailed the relationship between handgrip strength and various aspects of postoperative morbidity. Ten out of these 15 studies described a significant relationship between lower handgrip strength and postoperative morbidity [9,10,12,13,25,28,30-33]. No studies defined postoperative morbidity using validated morbidity tools. A range of morbidities were recorded prospectively: very few studies defined in detail how these morbidities were determined. Five studies used length of hospital stay as a surrogate for postoperative complications, but did not describe the associated morbidities.

**Table 5:** Type of Postoperative morbidity included in follow-up of patients.

	Pulmonary	Infectious	Renal	Gastrointestinal	Cardiovasular	Neurological	Wound	Haematological	Pain	LOS
**Mahalakshmi **[10]	Y	Y	Y	Y	Y	Y	Y			Sep

**Cook **[17]	Y	Y	Y		Y					Sep

**Figueiredo**[24]		Y						Y		Sep

**Le Cornu**[25]	Y	Y								Sep

**Guo**[11]		Y					Y			Sep

**Watters**[27]										Sep

**Schroeder**[34]										

**Griffith**[28]	Y	Y			Y					Y‡

**Brenner**[29]	Y	Y					Y			

**Webb**[30]										Y*

**Shukla**[31]	Y				Y		Y			

**Hunt**[13]	Y	Y	Y	Y			Y			Sep

**Davies**[32]										Y†

**Klidjian**[33]	Y	Y								Y*

**Klidjian**[9]										Y*

Y- Included in definition of complications. LOS(length of stay)-definition of morbidity includes length of stay greater than pre-defined level. Sep- LOS is analysed separately i.e. not included in definition of morbidity. Y* defined as complication if > 14 day LOS postop. Y† Complication resulting in ≥16 day LOS post op. Y‡ Defined as serious complication if > 14 day LOS.

### Length of Hospital Stay

Tables 5 and 6 show the 12 studies which utilised length of stay as an outcome measure for postoperative morbidity. Five of these studies incorporated length of hospital stay into their definition of "complications" [9,28,30,32,33] and 7 studies separately explored the relationship between handgrip strength and length of hospital stay [10,11,13,17,24,25,27]. Three of these 7 studies reported an association between lower handgrip strength and prolonged length of stay [10,13,25]. Mean or median values were compared rather than log-rank analysis.

**Table 6:** Studies describing relationship between Grip Strength (GS) and Hospital Length of Stay (LOS).

AUTHOR	GS of LOS "Controls" (kg or %)	Control LOS (mean days ± SD)	GS of LOS "comparators" (kg or %)	Comparator LOS (mean days ± SD)	Log-rank test?	LOS and Handgrip strength associated?
Mahalakshmi [10]	< 85%	12.8 ± 6.6	> 85%	9.3 ± 3.4	NO	YES

Cook [17]	Male < 32 kg Female < 20.5 kg	8.1 ± 10	Male > 32 kg Female > 20.5 kg	6.8 ± 7.5	NO	NO

Figueiredo [24]	ICU stay only*	Not presented	Not presented	Not presented	NO	NO

Le Cornu [25]	Not presented	Not presented	Not presented	Not presented	YES†	Positive correlation

Guo [11]	< 85%	42 ± 20	> 85%	32 ± 10	NO	

Watters [27]	Not presented	Not presented	Not presented	Not presented	NO	NO

Hunt [13]	< 85%	11.4 ± 12	> 85%	6.8 ± 3.8	NO	YES

Griffith [28]	Mean: Male 25.4 ± 9.1 kg Female 14.4 ± 4.3 kg	7/61 had "Complications" (definition included LOS > 14 days)	Mean: Male 30.2 ± 8.4 kg Female 14.9 ± 5.7 kg	48/61 had LOS < 14 days	NO	Not reported

Webb [30]	< 85%	20/51 had "complications" (definition included LOS > 14 days)	> 85%	7/39 had LOS < 14 days	NO	Not reported

Davies [32]	< 15 kg	27/37 had "Complications" (definition included LOS > 16 days)	> 15 kg	3/14 had LOS < 16 days	NO	Not reported

Klidjian [33]	< 85%	43/72 had "complications" (definition included LOS > 14 days)	> 85%	5/48 had LOS < 14 days	NO	Not reported

Klidjian [9]	< 85%	20/44 had "complications" (definition included LOS > 14 days)	> 85%	3/58 had LOS < 14 days	NO	Not reported

*Increased ITU stay was associated with lower handgrip strength (right (27 ± 6 and 36 ± 12 kg p < 0.01) and left (27 ± 7 and 35 ± 12 kg p = 0.01)

†There was a correlation between grip strength and day of discharge post-transplant (*r = *-0.41, *P *= 0.01). There was no association between grip strength and length of time spent on ventilatory support post-transplant (*r = *-0.250) or length of time spent on the intensive care unit post-transplant (*r *= -0.112)

* Survival plot from time of listing to transplant or death (not for grip strength).

### Mortality

Table 7 summarizes the 5 studies that explored the relationship between handgrip strength and postoperative mortality. Variable time points for postoperative associated death were defined across studies, ranging from 30 days to 6 month mortality following surgery. One study did not define the time period of follow-up for patients to determine mortality. Two studies reported an association between lower handgrip strength and increased mortality [12,17].

**Table 7:** Studies describing relationship between handgrip strength and postoperative mortality.

AUTHOR	YEAR	Duration of mortality follow-up	Mortality "Control" handgrip strength	Control Mortality	Mortality "comparator" Handgrip strength	Comparator Mortality	Log-rank?
Cook [17]	2001	3 months	Male < 32 kg Female < 21 kg	11.3%	Male > 32 kg Female > 21 kg	2.9%	NO

Figueiredo [24]^A^	2000	1 year	GS data not presented				n/a

Le Cornu [25]^B^	2000	30 days, 6 months	GS < 85%	Not reported	GS > 85%	Not reported	YES*

Griffith [28]	1989	7 days	Male 27 ± 6 kg Female 13 ± 4 kg	8.7% 13.3%	Figures not presented		NO

Kalfarentzos [12]	1989	Not stated	GS < 85%	17.2%	GS > 85%	0%	NO

GS = handgrip strength

A. None of the nutritional parameters assessed including handgrip strength were associated with increased risk of mortality

B. Survival plot from time of listing to transplant or death (not for grip strength)

*GS < 85% was significantly related to post transplant occurrence of major complications (definition includes death), minor sepsis and no sepsis (p = 0.05)

## Discussion

Contrary to large population studies, our systematic review of the relationship between preoperative handgrip strength and postoperative outcome did not find compelling data to support the hypothesis that the results of studies in the general population translate to perioperative medicine. The majority of studies were considered to be of reasonable quality. Despite these quality scores, many studies contained important potential confounding factors which varied markedly between studies. A range of different instruments have been employed to measure grip strength, with other corroborative assessments of strength being frequently absent. Due to the substantial variation in the way in which each specified outcome had been defined between studies, plus the lack of analyses testing any one particular association, it was not possible to perform meta-analyses of results or formally test the heterogeneity (consistency) between studies. This marked heterogeneity between studies limits any definitive conclusions for the perioperative environment and renders this preoperative assessment largely unexplored. Nevertheless, several of these studies - albeit with the limitations as discussed above - suggest the role for preoperative handgrip strength assessment should be explored further.

Large epidemiological studies have shown that perioperative morbidity is associated with dramatic differences in post-discharge life expectancy across different operations and health systems [2]. The cost and expertise required by certain preoperative tests, such as cardiopulmonary exercise testing, plus other limiting factors (e.g. dysmobility, acuity of surgery) necessitates an alternative approach to be developed for the objective assessment of perioperative risk in the substantial minority of patients who may sustain morbidity that impacts on their longer-term survival. The development of an inexpensive, mass screening preoperative assessment tool with high sensitivity and specificity to detect postoperative morbidity is clearly attractive. Handgrip strength is an easy, non-invasive, cheap, real-time and established independent "bedside" predictor of long-term all-cause mortality in more than 44,000 patients studied in the general population [14].

There are also compelling basic biological reasons for establishing the role of handgrip strength in preoperative assessment. Cardiopulmonary reserve is a long-established predictor of cardiovascular and all-cause mortality, in both asymptomatic individuals and patients with cardiovascular disease [35]. Cardiac insufficiency has emerged as the commonest preoperative morbidity associated with increased morbidity and mortality [36,37]. An important component of cardiac failure is dysfunctional skeletal muscle metabolism [38] and impaired strength - as reflected by handgrip strength [39]. Skeletal muscle exerts important effects on the patterns of substrate use during periods of increased cardiopulmonary performance [40,41]. Major alterations in skeletal muscle histology and biochemistry occur in patients with long-term heart failure [42,43]. These skeletal muscle adaptations may underlie the early onset of anaerobic metabolism, increased lactate production and fatigue in heart failure. Handgrip strength improves following specific interventions that increase cardiopulmonary reserve [44,45]. Muscle (handgrip) strength is also impaired in metabolic disease [46], which may in part explain its association with both poorer perioperative outcomes and all-cause mortality.

One limitation of this systematic review is that no original study data were retrieved, although given the heterogeneity of both study design and the surgical populations in question this would have been unlikely to alter the main conclusions. Because only published reports were examined (obtained from searches performed only on MEDLINE, EMBASE and Cochrane databases), a formal assessment of publication bias was not undertaken. It remains possible that not all relevant studies may have been identified since unpublished studies were not sought. There is very little perioperative demographic data provided in these studies, including cardiovascular risk and the identification of higher risk patients. Standards of postoperative care were not reported or apparently standardized. Since no interventions were conducted based on preoperative handgrip strength assessment, the studies only provide associative conclusions.

This systematic review has generated two significant clinical implications. Firstly, given the compelling general population data that predicts longevity, there is clearly a need for the further prospective assessment of whether preoperative handgrip strength can help stratify risk of adverse postoperative outcomes. Second, these studies demonstrate that handgrip strength is a feasible, pragmatic, real-time bedside tool that may enhance preoperative risk stratification.

## Conclusions

Impaired preoperative handgrip strength may be associated with increased postoperative morbidity, mortality and prolonged hospital stay following surgery. Given the robust predictive power of this inexpensive, objective bedside test beyond the perioperative population, further studies of its' role in predicting postoperative outcomes appear to be warranted provided prospective, objectively defined measures of morbidity are employed.

## Competing interests

The authors declare that they have no competing interests.

## Authors' contributions

All authors contributed to Study design, Conduct of study, Data analysis and Manuscript preparation.

## Pre-publication history

The pre-publication history for this paper can be accessed here:

http://www.biomedcentral.com/1471-2253/12/1/prepub

## Supplementary Material

Additional file 1 **Checklist of items demonstrating adherence to PRIMSA systematic review guidelines**. Click here for file

Additional file 2 **Newcastle Ottowa Scale**. Click here for file
